# Harnessing the Power of Technology to Transform Delirium Severity Measurement in the Intensive Care Unit: Protocol for a Prospective Cohort Study

**DOI:** 10.2196/62912

**Published:** 2025-09-25

**Authors:** Roshini Raghu, Keivan Nalaie, Ivan Ayala, Juan Jose Morales Behaine, Juan Pablo Garcia-Mendez, Hannah Friesen, Kathleen Leistikow, Anirban Bhattacharyya, Arun Jayaraman, Pablo Moreno Franco, Alejandro Rabinstein, Linda L Chlan, Malaz Boustani, Vitaly Herasevich, Heidi Lindroth

**Affiliations:** 1 Division of Nursing Research Department of Nursing Mayo Clinic Rochester, MN United States; 2 Division of Critical Care Department of Anesthesiology and Perioperative Medicine Mayo Clinic Rochester, MN United States; 3 Department of Anesthesiology and Perioperative Medicine Mayo Clinic Phoenix, AZ United States; 4 Department of Critical Care Medicine Mayo Clinic Phoenix, AZ United States; 5 Department of Critical Care Medicine Mayo Clinic Jacksonville, FL United States; 6 Department of Transplantation Medicine Mayo Clinic Jacksonville, FL United States; 7 Department of Neurology Mayo Clinic Rochester, MN United States; 8 Center for Health Innovation and Implementation Science School of Medicine Indiana University Indianapolis, IN United States; 9 Center for Aging Research School of Medicine Aurum Institute Indianapolis South Africa

**Keywords:** ICU delirium, intensive care unit, computer vision technology, passive digital marker, delirium severity, artificial intelligence, Alzheimer’s disease, other related dementias

## Abstract

**Background:**

Delirium, an acute brain dysfunction, is a complication in up to 50% of patients in the intensive care unit (ICU). Measuring and mitigating delirium severity can reduce associated morbidity and improve long-term health outcomes post discharge. However, the perceived complexity of the available delirium detection tools and clinical workload limits the routine assessment of delirium severity. Developing a passive digital marker for delirium severity, combining routine electronic health record (EHR) and computer vision technology data, could be an implementable, scalable, and sustainable approach.

**Objective:**

Our primary objective is to develop a passive digital marker for delirium severity (PDM-Del) and examine its performance in comparison to validated delirium severity tools. Our secondary objective is to evaluate the acceptability and usability of the PDM-Del by patients, families, and clinicians.

**Methods:**

We will conduct a prospective, longitudinal cohort study to develop a PDM-Del using computer vision data and routinely collected EHR data. Following informed consent, the study team will collect image data through continuous digital video recordings of adult patients (>50 years) in their ICU room, routine EHR data (demographic and clinical variables), and administer delirium severity assessments (4 times daily) until ICU discharge or death. We will examine the usability and acceptability of the developed PDM-Del by patients, families, and direct care clinicians in a pilot randomized controlled clinical trial (aim 3). Descriptive statistics (means, SDs, medians, IQRs, and frequencies) and statistical differences between study instruments will be examined. We will use convolutional neural networks and machine learning to inform model development, testing, and validation. We will report model performance statistics, including accuracy, precision, recall, and the *F*_1_-score.

**Results:**

We are currently in the recruitment and data collection phase. As of March 2025, we screened 3980 patients (32% eligible, n=1307), approached 665 (50%), and enrolled 150 participants (23% enrollment rate). Among the 150 patients, the median age was 67 (IQR 61-74) years, 62% (93/150) were male, and 91% (136/150) were White.

**Conclusions:**

The PDM-Del could provide real-time, actionable feedback to direct care clinicians on the brain health of patients in the ICU. Early mitigation of delirium severity may decrease the risk of mortality, future Alzheimer disease and related dementia, and length of hospital stay.

**Trial Registration:**

ClinicalTrials.gov NCT06172491; https://clinicaltrials.gov/study/NCT06172491

**International Registered Report Identifier (IRRID):**

DERR1-10.2196/62912

## Introduction

### Background

Delirium, an acute brain dysfunction that manifests as a disturbance in attention (ie, reduced ability to direct, focus, sustain, and shift attention), accompanied by reduced awareness of the environment, is seen in up to 50% of mechanically ventilated patients in the intensive care unit (ICU) [1]. Delirium increases the ICU and hospital length of stay, leading to an estimated annual cost of US $152 billion [2-7]. Furthermore, delirium occurrence has been associated with an increased risk of future Alzheimer disease and related dementia (ADRD), with 26% of delirium survivors developing cognitive scores closer to mild ADRD at a 12-month evaluation [8]. Previous studies report that not only the occurrence of delirium (presence or absence) but also its severity increases the risk of mortality, institutionalization, and ADRD [3,9-13]. Delirium severity trajectories have been independently associated with increased 30-day mortality [9]. Increasing levels of delirium severity are also associated with decreased odds of discharge to home (multivariable; odds ratio [OR] 0.78, 95% CI 0.71-0.86) and a faster rate of global cognitive decline in a postoperative population [3,10]. Measuring delirium severity is a powerful prognostic tool associated with important health outcomes. In addition to prognostication, the longitudinal and sequential measurement of delirium severity tracks the patients’ response to mitigation strategies over time.

**Figure 1:**
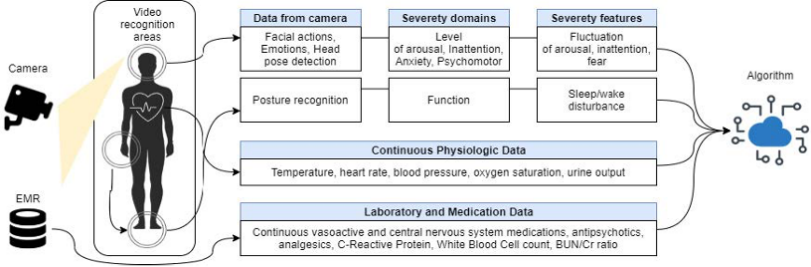
Mapping the outline of developing an artificial intelligence algorithm through computer vision technology and electronic health records data. BUN: blood urea nitrogen; Cr: creatinine; EMR: electronic medical record.

Despite the existence of validated tools for measuring delirium severity, it is not regularly measured in clinical care, and up to 70% of cases of delirium are missed in routine ICU care [14-16]. These gaps in clinical care could be attributed to clinician workload, staff turnover, and the perceived complexity or lack of knowledge on using current delirium monitoring tools [16-20]. A recent study assessing nurses’ perceptions of delirium assessment tools reported that nurses preferred scaled tools over binary instruments as a scaled score supports the monitoring of the patients’ response to mitigation strategies [21]. Implementing frequent or continual documentation of minute-by-minute or hour-by-hour observations required for accurate delirium severity measurement is not feasible as it adds to the existing documentation burden faced by direct care clinicians [22,23]. One solution to address these limitations is to incorporate advanced artificial intelligence (AI) approaches, such as computer vision technology (CVT), in delirium care. CVT prospectively collects continuous observation (streaming) data that is collated and condensed for human decision-making [23-27]. Key features of delirium severity collected primarily through observation of the patient’s behavior are prime candidates for continuous measurement by CVT, as outlined in Figure 1.

Recent studies have demonstrated the feasibility of CVT use in the ICU, including delirium detection [23,24,27-29]. Compared to patients without delirium (n=8), those with delirium (n=4) demonstrated significantly different facial actions (eg, lip, cheek, and nose wrinkling), facial expressions (eg, sadness, pain, fear, and disgust), head pose and movement, and posture (eg, lying, sitting, and standing) [23]. This pilot study provided foundational work to investigate further how continuous, noncontact CVT data can measure delirium severity in the ICU. While CVT data may be sufficient to capture some features of delirium severity, the model performance may improve with the addition of routinely collected electronic health record (EHR) data such as medications and physiologic response (illness severity). Therefore, we seek to address this gap in research and clinical care by developing a passive digital marker for delirium severity (PDM-Del) that combines relevant CVT data with existing routine EHR data to produce a hybrid PDM that automatically and passively measures and monitors delirium severity.

### Study Aims

The objective of the study had 3 aims:

1. To train convolutional neural networks (CNNs) to measure delirium severity using prospective observational computer vision data in older adults (age >50 years) in the intensive care unit.
2. To develop a PDM-Del using prospectively collected computer vision data and/or routine EHR data in a cohort of aging adult patients in the ICU.
3. To determine the usability and acceptability of the passive digital marker for delirium severity in a prospective pilot randomized controlled clinical trial in aging adult patients in the ICU.

## Methods

### Study Design

This prospective longitudinal study is recruiting critically ill, aging adults (age ≥50 years) admitted to ICUs at the designated study sites. We obtained institutional review board approval from Mayo Clinic (IRB), and the principal investigator (HL) registered the trial on ClinicalTrials.gov (NCT06172491). The study team uses Agile methodologies to conduct the day-to-day operations of the study. The study started recruiting on January 1, 2023.

### Ethical Considerations

We submitted the study for human participant ethics review board during IRB submission and obtained approval prior to the start of the study (IRB#22-003098, Mayo Clinic). Study participants do not receive any compensation for participation in the study.

#### Informed Consent

The study team obtains informed consent for study participation from the patient or the identified proxy decision maker, with the option to opt out at any time. Participants provide specific permissions to share patient health information, including the use of video images in future presentations, for use in future research, and with external collaborators. IRB-approved study personnel approach eligible patients for inclusion into the study. Delirium status assessments are conducted before obtaining consent. If a potential patient is determined to have delirium, dementia, or a decreased level of arousal (RASS [Richmond Agitation and Sedation Scale] <–1 or >+1), proxy decision makers are approached for consent (ie, legally authorized representative). Once a consented participant regains capacity, the study team confirms the continued study participation with the participant.

#### Privacy and Confidentiality

Only IRB-approved study personnel can access study information and collected patient data. Following informed consent, participants are assigned a unique study ID to maintain confidentiality. The consent document and associated personal health information, such as name and medical record number, are stored in a separate folder from associated study data that are maintained in a password-protected folder on an encrypted computer behind institutional firewalls. The EHR and delirium assessments collected by the study team are de-identified. The collected videos rely on facial detection and therefore cannot be deidentified. These videos (ie, images) are maintained on a password-protected, encrypted computer that is only accessible by IRB-approved study personnel.

### Inclusion and Exclusion Criteria

#### Inclusion Criteria

Eligibility criteria include older adults (age ≥50 years) with an estimated length of stay ≥24 hours in the ICU (including mechanically ventilated patients). To establish usability and acceptability, adult nurse clinicians (≥18 years), employed by the involved institutions, and assigned to care for a consented study patient for >4 hours, and adult proxy decision makers for patient participants (>18 years) who are willing to complete usability and acceptability surveys will be recruited for study participation.

Adult patients in the ICU ≥18 years of age are at an increased risk of delirium following an ICU admission. We chose the cutoff of 50 years of age or older to balance the increased risk from ICU admission along with the increased risk that accompanies aging adults. Each patient can function as their own case study with a unique set of observations; therefore, an ICU stay of 24 hours or greater was set as a temporal inclusion criterion.

#### Exclusion Criteria

Exclusion criteria include patients admitted for acute alcohol intoxication, drug (prescribed or illicit) overdose or withdrawal, acute neural injury, and unable to communicate with the research team due to sensory deficits (aphasic, blind, or deaf) or language issues (requires interpreter). Patients who were admitted for acute neurologic injury, alcohol withdrawal, or drug overdoses are excluded, as these injuries demonstrate similar behavioral observations to delirium. Patients with severe sensory impairments are excluded as delirium severity assessments currently require verbal and auditory-dependent patient participation.

To assess for usability and acceptability, nurse clinicians not assigned to the study participants and proxy decision makers who did not visit the patient in the ICU during the study period are excluded.

### Instruments and Data Collection

Longitudinal data collection (observational, n=400) includes continuous digital video images of the patient in their ICU room, routine EHR data, and delirium severity assessments collected by the study team to be used for the development of the convolutional neural network (aim 1) and the future passive digital marker for delirium severity.

For assessing sustainability and acceptability, we will collect data using the System Usability Scale (SUS), the Mayo Clinic Acceptability survey, and the Treatment Acceptability and Preferences (TAP) questionnaire. We will include data from enrollment until death or discharge from the ICU.

#### CVT Data

High-resolution and wide field-of-view computer vision data are collected 24/7 through video cameras for a minimum of 72 hours or until discharge or death, whichever occurs first. The cameras are positioned to capture the patient’s face, their body (all 4 limbs), and the overall patient room. A touchscreen user-friendly interface allows clinical staff, families, or patients to stop and restart recording. The observational data collected through video images will be used to perform face detection, facial expression, head pose detection, posture recognition, vital sign waveform data, and disturbance in the sleep-wake cycle as outlined in Textbox 1. Examples of open-source algorithms are provided in Textbox 1. These are provided for reference; however, the study is not committed to using these specific open-source algorithms.

**Computer vision data (images) collected may be processed using the following open-source algorithms (provided as examples).**

Description of proposed analysis of computer vision data

**Face detection**

FaceNet algorithm, Inception-ResNet V1 model. This is a publicly available open-source code developed by Google [30]. The model will detect the patient’s face as a foundational step to completing other measurements, such as emotion detection.

**Facial action unit**

OpenFace deep neural network 2.2.0 toolbox to detect facial action units (n~action units) [31].

Includes eyebrows (upper, lower, and blink) and lip movement. Coded as present (yes/no) and graded in intensity (0-5). Open-source, publicly available code.

**Facial expression**

Facial Action Coding System with OpenFace deep neural network 2.2.0 toolbox [31]. Eight common facial emotions. Frequency and length calculated. Anger, contempt, disgust, fear, happiness, pain, sadness, and surprise.

**Head pose detection**

OpenFace deep neural network 2.2.0 toolbox [31]. Detects movement and position of head (rotation, up/down movements, and side-to-side).

**Eye/Gaze Tracking**

OpenFace deep neural network 2.2.0 toolbox [32].

**Posture recognition**

OpenPose multiperson estimation model [33]. Localizes anatomical key points of limbs/joints, length of limbs/angles, and informs recognition of movements and activity. Will calculate speed and absence of movement (including presence of chemical or physical restraints). Open-source, publicly available code.

**Sleep-wake cycle**

Posture recognition will measure patient posture, movement, and sleep poses.

**Acute onset/fluctuation**

Acute onset will be defined as an abrupt change in any of the computer vision technology features.

Fluctuation will be defined as the % variation in any of the computer vision technology features.

**Cameras**

Each intensive care unit room will begin with 1-3 video cameras to investigate which camera positions/angles are necessary to capture the outlined information. The necessity of >1 camera will be continually reviewed.

#### EHR Data

Patient demographic and clinical variables pertaining to the current ICU stay will be extracted from the EHR. Textbox 2 outlines these data. The variables selected for inclusion are informed by literature [34-36]. EHR data is automatically extracted every 15 minutes and stored in the ICU DataMart for all Mayo Clinic locations [37]. For the Delirium Severity feature, we will extract the RASS score and sedation administration to assess for the fluctuating course and medication administration to assess for psychomotor disturbance.

**Routine electronic health record data to inform a passive digital marker for delirium severity.**

Data collected

**Illness severity**

- Vital sign (minimum, maximum, and range)
- Vasopressor use
- Anti-infective use
- SOFA (Sequential Organ Failure Assessment Score) score [38]

**Delirium severity**

- RASS (Richmond Agitation and Sedation Scale) score (minimum, maximum, and range)
- Propofol use
- Dexmedetomidine use
- Benzodiazepine use
- Opioid use
- Paralytics use
- Benzodiazepine use
- Antidepressant use
- Sleep enhancement (eg, melatonin)
- Antiseizure use
- Neurological assessment scales

**Covariates**

- Age
- Sex
- Ethnicity
- Race
- Past medical history

#### Standardized Delirium Severity Measurement

The study team has been trained by the principal investigator (HL) to administer standardized, validated delirium severity assessments outlined in Textbox 3, four times daily between 5 AM and 10 PM until death or discharge from the ICU. An observational field study accompanies tool administration and uses a standardized data collection form to promote interrater reliability. Observations include behaviors, emotions, head, eye, and limb movement, or the absence of these items. The study team timestamps the CVT data images when assessments are performed to assist with labeling and annotation. Concurrent to delirium severity assessments, the study team asks the assigned direct care nurse about their concern for the patient’s condition. Their concern is measured using the Worry Factor score, a 5-level scale with 0 being no concern and 4 being extreme concern [39]. The Worry Factor score was developed at the Mayo Clinic to improve early detection of acute deterioration.

**Delirium and delirium severity assessment administered 4 times daily between 5:00 AM and 10:00 PM.**

Delirium assessment tools and their definitions

**CAM-ICU (Confusion Assessment Method for ICU [intensive care unit])** [40,41]

Gold standard. Scored as positive or negative. Sensitivity 85% and specificity 87%

**ICDSC (Intensive Care Delirium Screening Checklist)** [42,43]

Validated intensive care unit tool. Score (0-8) on symptom presence. Sensitivity 95% and specificity 91%

**CAM-ICU-7 (Confusion Assessment Method for the Intensive Care Unit–7-point scale)** [42,44]

Validated delirium severity tool scored directly from CAM-ICU, Cronbach α=0.85. Scoring (0-7): 0-2=no delirium, 3-5=mild/moderate delirium, 6-7=severe delirium.

**DRS-R-98 (Delirium Rating Scale-R-99)** [45,46] (nonintubated only)

Validated delirium severity tool for nonintubated patients. 16-item (0-44 score) symptom/feature measurement. Cronbach α=0.90

The PMD study (principal investigator: MB) and the Better Assessment of Illness study findings guided the selection of these tools [43,44,47,48]. Scales and subcomponents of each scale will be applied as a continuum of delirium severity to train the CVT neural networks.

To assess usability and acceptability (pilot randomized controlled trial, n=30), we will enroll one patient at a time across ICUs with similar patient populations and delirium rates to reduce the risk of contamination and randomize (1:1, computer-generated assignment) to usual care (nurse-administered Confusion Assessment Method for the Intensive Care Unit, standard care plan in place) or intervention (PDM-Del score, suggested interventions for level of severity; eg, mild=reorientation). The patient, proxy decision maker, and direct care clinicians assigned to care for the patient will complete acceptability and usability assessments: SUS, Mayo Clinic Acceptability survey, and the TAP questionnaire. To reduce learning effects by nurses, we will consider block randomization at the institutional level instead of randomization at the patient level. Table 1 outlines data collection for aim 3.

**Table 1:** Usability and acceptability of passive digital marker for delirium severity.

Tools and their description		Participant and time of completion
**System Usability Scale** [49]		
	10 questions rate usability on a 1-5 scale, strongly disagree-strongly agree	Nurse clinician and end of shift with the study patient
**Mayo Clinic Acceptance Survey** [28]		
	10 questions on the use of computer vision technology in ICU^a^, Likert scale 1-5	Nurse clinician and end of shift with the study patient
**Treatment Acceptability and Preferences Questionnaire** [50]		
	4 questions, Likert scale on effectiveness, believability, and acceptability of PDM-Del^b^, high internal consistency (α>.80)	Patient and identified proxy and at ICU discharge
**EHR^c^ data**		
	Compliance with A2F^d^ bundle [51], time to extubation, ICU mortality	Extracted from the EHR at ICU discharge and secondary outcomes

^a^ICU: intensive care unit.

^b^PDM-Del: passive digital marker for delirium severity.

^c^EHR: electronic health record.

^d^A2F: A: assess, prevent, and manage pain; B: both spontaneous awakening trials and spontaneous breathing trials; C: choice of analgesia and sedation; D: delirium (assess, prevent, and manage); E: early mobility and exercise; F: family engagement and empowerment.

#### Usual Care Group

In the usual care group, nurse administration of the Confusion Assessment Method for the Intensive Care Unit delirium assessment will be done once every 12 hours along with the administration of evidence-based strategies to mitigate delirium, such as the A2F bundle (ie, usual care).

#### Intervention Group

In the intervention group, the PDM-Del score will be displayed on a monitor in the patient’s ICU room for the nurses' (formal caregiver) reference. Evidence-based strategies to mitigate delirium severity, such as the A2F bundle, will be suggested underneath the score for the nurse clinician to apply. Nurses will be asked to provide feedback on whether they felt the intervention suggested was appropriate. These are the initial steps to develop a decision support tool for nurses to mitigate delirium severity.

### Statistical Analysis

#### Power

The proposed sample size was calculated to develop the algorithms specified in aims 1 and 2. We assumed a delirium rate (prevalence and incidence) of 45% in enrolled patients as reported in recent literature [52,53]. HL recognizes the likelihood that this study may be underpowered. These findings will inform future grant applications to adequately power study aims. The minimum computer vision frames (n=2000) needed per class label (200 estimated labels; 80 CVT features and 120 delirium severity observations) were used to calculate the sample size [23]. Assuming an average of 2 frames per second and an average of 48 hours of video recording per patient, ~140 patients will be needed to train the CNN to measure delirium severity. An additional ~260 patients will be recruited to develop a PDM for a total sample of 400. This is powered for a confidence level of 0.95, an interval width of 0.10, and the ability to detect a 0.80 area under the curve. An additional 30 patients will be recruited for the pilot randomized controlled trial to determine the usability and acceptability of the PDM. Effect sizes will power future R01 or R-series grant applications designed to further test and refine the PDM-Del.

#### Data Analysis

**Figure 2:**
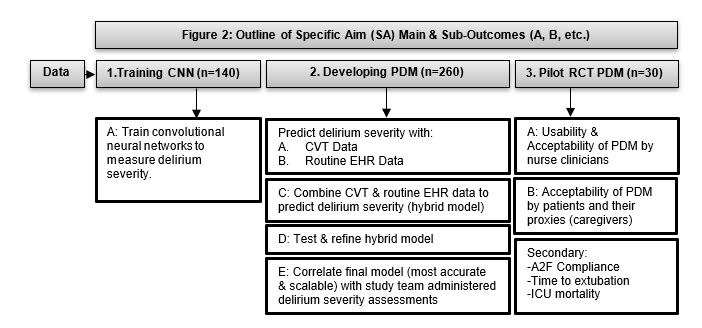
Outline of specific aims, main outcomes, and suboutcomes (A, B, etc). A2F=ABCDEF Bundle: A: assess, prevent, and manage pain; B: both spontaneous awakening trials and spontaneous breathing trials; C: choice of analgesia and sedation; D: delirium (assess, prevent, and manage); E: early mobility and exercise; F: family engagement and empowerment; CNN: convolutional neural network; CVT: computer vision technology; EHR: electronic health records; ICU: intensive care unit; PDM: passive digital marker; RCT: randomized controlled trial.

Descriptive statistics (means, SDs, medians, IQRs, and frequencies) will be reported. Data among sites will be examined for statistical differences. Modeling assumptions (dependent on the types of models used) will be verified [23,27,29,54]. Missing data will be examined for level of missingness (missing completely at random, missing at random, etc) and procedures such as multiple imputation completed as needed. Figure 2 provides an overview of the planned statistical analysis.

Data at the patient level will be split randomly 80/20 for training and testing purposes. Labeling and annotation of the CV images to train the CNNs will follow a stepwise process. Active learning is being used to minimize the number of manual annotations needed for modeling procedures. The annotation process will be iterative and focused on specific problems such as object recognition. When annotations are needed, a 2-step process that incorporates quality control checks and interrater reliability metrics will be implemented. Both supervised and unsupervised learning will be considered. Classification accuracy, model performance data including *F*_1_-scores, correlations between the final CNN model, and administered delirium severity assessments (ordinal scale), will be evaluated and reported.

Exploratory analysis, factor association measurement, and multiclass machine learning models and ensembles (eg, random forests, gradient boosting, bagging methods, and support vector machines) will inform analysis. Depending on the number of delirium severity classes (ie, mild, moderate, and severe) identified, classification probabilities will be calculated. Classification into one of the severity classes will be based on the class with the highest predicted probability. Test error, variance, entropy, inference, Floating Point Operations Per Second, number of trees, and depth of forest statistics will inform model selection. Accuracy and precision of classification will evaluate model performance. Nested cross-validation will evaluate the final models, perform hyperparameter searches, and estimate the generalization error of the underlying models. Performance statistics (ie, sensitivity and specificity), including *F*_1_-scores, will be reported. The Youden Index will determine optimal cut-off points for mild, moderate, and severe delirium [55]. ICU outcomes (admission and discharge time, mortality, discharge disposition, 30-day readmission) associated with the models will be reported for internal validation. We will select the model that balances accuracy with scalability for future testing and evaluation. A separate dataset of new patients will be used for external validation and performance evaluation of the model.

We will calculate means, SDs, and 90% confidence intervals for the SUS, the Mayo Clinic Acceptability survey, and the TAP questionnaire. We hypothesize that the PDM-Del will be acceptable and usable by frontline ICU nurse clinicians, patients, and identified proxies. The secondary outcomes of A2F bundle compliance, time to extubation, and ICU mortality will be collected and compared between groups [51]. Generated effect sizes will power future R01 grant applications.

## Results

As of January 2023, data collection began and is ongoing across 3 health care institution sites. We have screened 3,980 patients, approached 665, and enrolled 150 participants as of March 2025. Among the 150 patients, the median age was 67 (IQR 61-74) years, 62% (93/150) were male, and 91% (136/150) were Caucasian. More than 34 million images were collected, and more than 1500 delirium severity assessments were completed.

The primary reason patients declined participation in the study to date was feeling uncomfortable with the continuous image collection and the video camera. Secondary reasons to decline study participation included patients not being interested in research while in the ICU, and finding the study team assessments (4 times daily delirium assessments) exhausting. To address these limitations in study recruitment, the principal investigator has presented to formal community groups and the study’s data safety monitoring board to gain insight into how to improve the study. As a result, the study team is moving the camera to a different location in the room and has developed a frequently asked questions flyer for patients and families to reference.

The main inclusion criteria that were not met, leading to ineligible patients, was the length of stay of 24 hours or greater in the ICU (29.1%), followed by acute neurological injury (14.9%). Additional reasons for slower recruitment than expected are equipment malfunctions and availability, study team vacancies, and the length of the onboarding to study protocol.

## Discussion

### Overview

Delirium, while currently categorized as present or absent, lacks granularity in assessing severity [56]. While this is sufficient for detection and diagnosis, it may be better to obtain information on the level or trajectory of delirium severity. The passive digital marker for delirium severity (PDM-Del) aims to address this limitation and provide immediate and actionable information to healthcare clinicians on the patient’s brain health, similar to other vital signs like heart rate. As clinicians work to mitigate delirium severity, the PDM-Del would provide actionable and nonjudgmental feedback on the interventions’ effectiveness, providing the opportunity to individualize care based on the patient’s response. The underlying hypothesis of this work is that clinicians will take more action to mitigate delirium severity if they receive prompt and actionable feedback on the performance of their interventions in real time. This study presents a novel approach to measuring delirium severity through the development of a scalable, feasible, and passive digital marker.

Early recognition and mitigation of severity play a crucial role in preventing adverse health outcomes and enhancing the lives of critically ill adults. The proposed PDM-Del may improve the lives of patients in the ICU by providing timely and actionable feedback to bedside clinicians about delirium severity, supporting the mitigation of delirium severity. In turn, the risk of future mortality and morbidities, including ADRD, associated with delirium severity may be reduced [8]. The close association between delirium and dementia has been studied exclusively, with a possible synergistic interrelationship between the two conditions [57-59]. Hospitalized patients with ADRD are at least twice as likely to develop delirium superimposed on dementia, and patients who develop in-hospital delirium are at a higher risk of subsequently developing dementia in the future. A prospective study on hospitalized patients with ADRD reported that 33% had delirium superimposed on dementia, and 25% of those patients with delirium superimposed on dementia had an increased mortality rate, functional decline, and increased hospital length of stay [60]. Delirium superimposed on dementia can be challenging to detect in the clinical setting due to the overlapping cognitive and behavioral features between dementia and delirium [61]. Yet, it is critical to detect delirium in this population as it likely signifies a medical emergency. We aim for our PDM-Del to be a valuable tool to improve the early recognition of delirium superimposed on dementia. Future studies may also examine how computer vision could aid in the recognition of symptoms such as pain and anxiety in patients with ADRD. Focusing on this vulnerable patient population may help reduce their inpatient and posthospitalization morbidity and mortality and improve their quality of life.

Clinical research to identify delirium subtypes and biological biomarkers could be accelerated with the use of a PDM-Del. Instead of relying on inconsistent clinical assessments or resource-intensive research assessments, the PDM-Del would provide continuous monitoring and real-time measurement across a large population of critically ill patients. The availability of such data could greatly expand and expedite the use of data science methods such as machine learning, elucidating biological and clinically relevant relationships that, in turn, could improve clinical trials aimed at identifying effective treatments for delirium. Furthermore, the PDM-Del can be used to study the trajectories of delirium severity and identify clinical interventions to modify the trajectory in real time, therefore potentially mitigating adverse outcomes such as post–intensive care syndrome [9].

The ICU of the future is poised to harness advanced AI technologies and ambient monitoring, specifically CVT, to augment patient monitoring, assessment, and support clinical decision-making. Recent studies show that CVT collected through ambient sensors in the ICU can perform autonomous patient monitoring, quantify progress in patients’ mobility, monitor safety compliance, and detect differences between patients with and those without delirium [23-25,27,28,32,62]. This type of ambient CVT operates at a low variable cost, is passive, fatigue-free, and ceaseless, thus ideal for continuously measuring delirium severity. Depending on the performance of CVT to automatically measure delirium severity, additional ambient sensors may be considered in future studies to improve model performance and/or develop a more scalable and sustainable passive digital marker. These ambient sensors include passive infrared motion sensors, bed sensor devices that measure sleep quality, ambient vital sign monitors that measure continuous heart rate, respiratory rate, and thermography are a few examples that could be used to automatically measure delirium severity and provide feedback for delirium mitigation strategies [63].

Upon completion of the study aims, the machine learning methods used to integrate various data modalities, develop data pipelines, and model architectures will be reported. We will partner with direct care clinicians and use user-centered design principles to codesign the PDM-Del score structure, interface, and clinical integration. This work will be detailed in subsequent manuscripts. Future research is needed to explore how clinicians will interpret and integrate the PDM-Del score into daily practice. While we recognize that the scientific community may prefer the term "passive digital marker", we have opted to name the algorithm "BrainSaver" for use by clinicians." As we progress through usability and clinical deployment stages, we will work with the end user (clinicians, patients, families) to identify an appropriate name.

### Limitations

The critical care setting presents unique challenges to autonomous delirium severity measurement, including paralyzed, mechanically ventilated, deeply sedated patients and the use of physical or chemical restraints. The impact of sedation levels on facial actions and expressions remains insufficiently documented. Therefore, the study team will closely document the level of sedation and the use of restraints in study participants to investigate this further.

It has been noted that privacy concerns play a significant role in limiting recruitment to our study. Since recruitment began, the most common reason for denied participation in the study is because the patients or families do not want to be recorded, followed by the discomfort in taking part in research while at the ICU. To address this issue, HL met with the Health Data and Technology Advisory Board, where the first author (RR) presented a mock consent and received feedback on the approach toward the consenting process. Study investigators (HL and VH) are also meeting with the ICU health care teams and patient partners to address these concerns and will continue to do so throughout the proposed project. In terms of analyzing computer vision data, we have identified that the collection of minimal amounts of information is needed to train and use the model so that patients’ privacy and safety are protected. This includes blurring or removing unnecessary pieces of data that do not contribute to model development. To address staff concerns with the use of computer vision technology, the principal investigator and study team presented at unit meetings, held lunch and learn events, and recruited nurse champions from each participating unit to proactively learn and address privacy concerns. These efforts continue as the work is ongoing.

We have also encountered unforeseen challenges in the labeling and training of computer vision data that hinder progress. To optimize data collection through camera placement, the study team frequently uses the ICU simulation center to conduct experiments on camera placement, angle, aperture, and lighting.

This study is the first study to our knowledge that aims at creating a PDM-Del for continuous delirium severity measurement. Our study proposes a solution to the identification of missed delirium cases in critical care practice so that patients receive early interventions for delirium mitigation. As our assessments also encompass delirium severity, patients with severe or moderate delirium can be identified early, and specific behaviors related to these severities can be studied in detail. The results of the proposed study will be reported in full transparency, and data will be shared when applicable. The relevant biological variables of assigned sex and age will be used as covariates.

### Conclusion

This study aims to develop a passive digital marker for delirium severity using CVT, a form of AI, combined with routine electronic health record data. By combining two different data modalities enhanced with AI, we anticipate a significant clinical impact. The continuous measurement of delirium severity, akin to a vital sign, is expected to prompt clinicians to take early and frequent actions for delirium mitigation. Through early recognition and mitigation of delirium severity, the proposed PDM-Del may decrease the risk of an extended hospital length of stay, ADRD, mortality, and improve the quality of life of ICU patient survivors and families.

## Abbreviations

AI: artificial intelligence
ADRD: Alzheimer disease and other related dementia
CNN: convolutional neural network
CVT: computer vision technology
EHR: electronic health record
ICU: intensive care unit
IRB: institutional review board
PDM-Del: passive digital marker for delirium severity
RASS: Richmond Agitation and Sedation Scale
TAP: Treatment Acceptability and Preferences Questionnaire
SUS: System Usability Scale

## References

[1] (2022). Diagnostic and Statistical Manual of Mental Disorders (DSM-5-TR).

[2] Ha A, Krasnow RE, Mossanen M, Nagle R, Hshieh TT, Rudolph JL, Chang SL (2018). A contemporary population-based analysis of the incidence, cost, and outcomes of postoperative delirium following major urologic cancer surgeries. Urol Oncol.

[3] Khan B, Perkins AJ, Gao S, Hui SL, Campbell NL, Farber MO, Chlan LL, Boustani MA (2017). The confusion assessment method for the ICU-7 delirium severity scale: A novel delirium severity instrument for use in the ICU. Crit Care Med.

[4] Gleason LJ, Schmitt EM, Kosar CM, Tabloski P, Saczynski JS, Robinson T, Cooper Z, Rogers SO, Jones RN, Marcantonio ER, Inouye SK (2015). Effect of delirium and other major complications on outcomes after elective surgery in older adults. JAMA Surg.

[5] Vasilevskis EE, Chandrasekhar R, Holtze CH, Graves J, Speroff T, Girard TD, Patel MB, Hughes CG, Cao A, Pandharipande PP, Ely EW (2018). The cost of ICU delirium and coma in the intensive care unit patient. Med Care.

[6] Leslie DL, Marcantonio ER, Zhang Y, Leo-Summers L, Inouye SK (2008). One-year health care costs associated with delirium in the elderly population. Arch Intern Med.

[7] Leslie DL, Inouye SK (2011). The importance of delirium: economic and societal costs. J Am Geriatr Soc.

[8] Pandharipande P, Girard T, Jackson J, Morandi A, Thompson J, Pun B, Brummel N, Hughes C, Vasilevskis E, Shintani A, Moons K, Geevarghese S, Canonico A, Hopkins R, Bernard G, Dittus R, Ely E, BRAIN-ICU Study Investigators (2013). Long-term cognitive impairment after critical illness. N Engl J Med.

[9] Lindroth H, Khan BA, Carpenter JS, Gao S, Perkins AJ, Khan SH, Wang S, Jones RN, Boustani MA (2020). Delirium severity trajectories and outcomes in ICU patients. Defining a dynamic symptom phenotype. Ann Am Thorac Soc.

[10] Vasunilashorn SM, Fong TG, Albuquerque A, Marcantonio ER, Schmitt EM, Tommet D, Gou Y, Travison TG, Jones RN, Inouye SK (2018). Delirium severity post-surgery and its relationship with long-term cognitive decline in a cohort of patients without dementia. J Alzheimers Dis.

[11] Andrews PS, Wang S, Perkins A J, Gao S, Khan S, Lindroth H, Boustani M, Khan B (2020). Relationship between intensive care unit delirium severity and 2-year mortality and health care utilization. Am J Crit Care.

[12] Rosgen B, Krewulak KD, Stelfox HT, Ely EW, Davidson JE, Fiest KM (2020). The association of delirium severity with patient and health system outcomes in hospitalised patients: a systematic review. Age Ageing.

[13] Girard TD, Thompson JL, Pandharipande PP, Brummel NE, Jackson JC, Patel MB, Hughes CG, Chandrasekhar R, Pun BT, Boehm LM, Elstad MR, Goodman RB, Bernard GR, Dittus RS, Ely EW (2018). Clinical phenotypes of delirium during critical illness and severity of subsequent long-term cognitive impairment: a prospective cohort study. Lancet Respir Med.

[14] Steis MR, Fick DM (2008). Are nurses recognizing delirium? A systematic review. J Gerontol Nurs.

[15] Spronk PE, Riekerk B, Hofhuis J, Rommes JH (2009). Occurrence of delirium is severely underestimated in the ICU during daily care. Intensive Care Med.

[16] Pisani MA, Devlin JW, Skrobik Y (2019). Pain and delirium in critical illness: an exploration of key 2018 SCCM PADIS guideline evidence gaps. Semin Respir Crit Care Med.

[17] Devlin JW (2008). Assessment of delirium in the intensive care unit: nursing practices and perceptions. American Journal of Critical Care.

[18] Devlin JW, Bhat S, Roberts RJ, Skrobik Y (2011). Current perceptions and practices surrounding the recognition and treatment of delirium in the intensive care unit: a survey of 250 critical care pharmacists from eight states. Ann Pharmacother.

[19] Oxenbøll-Collet M, Egerod I, Christensen V, Jensen J, Thomsen T (2018). Nurses' and physicians' perceptions of confusion assessment method for the intensive care unit for delirium detection: focus group study. Nurs Crit Care.

[20] Rowley-Conwy G (2018). Barriers to delirium assessment in the intensive care unit: A literature review. Intensive and Critical Care Nursing.

[21] Nielsen AH, Larsen LK, Collet MO, Lehmkuhl L, Bekker C, Jensen JF, Laerkner E, Nielsen TA, Rossen BS, Thorn L, Laursen E, Fischer S, Villumsen M, Shiv LH, Høgh M, Rahr MN, Svenningsen H (2023). Intensive care unit nurses' perception of three different methods for delirium screening: A survey (DELIS-3). Aust Crit Care.

[22] Collins S, Couture B, Kang MJ, Dykes P, Schnock K, Knaplund C, Chang F, Cato K (2018). Quantifying and visualizing nursing flowsheet documentation burden in acute and critical care. AMIA Annu Symp Proc.

[23] Davoudi A, Malhotra KR, Shickel B, Siegel S, Williams S, Ruppert M, Bihorac E, Ozrazgat-Baslanti T, Tighe PJ, Bihorac A, Rashidi P (2019). Intelligent ICU for autonomous patient monitoring using pervasive sensing and deep learning. Sci Rep.

[24] Greco M, Caruso PF, Cecconi M (2021). Artificial intelligence in the intensive care unit. Semin Respir Crit Care Med.

[25] Milstein A, Topol EJ (2020). Computer vision's potential to improve health care. Lancet.

[26] Pickering BW, Litell JM, Herasevich V, Gajic O (2012). Clinical review: the hospital of the future - building intelligent environments to facilitate safe and effective acute care delivery. Crit Care.

[27] Yeung S, Rinaldo F, Jopling J, Liu B, Mehra R, Downing NL, Guo M, Bianconi GM, Alahi A, Lee J, Campbell B, Deru K, Beninati W, Fei-Fei L, Milstein A (2019). A computer vision system for deep learning-based detection of patient mobilization activities in the ICU. NPJ Digit Med.

[28] Glancova A, Do QT, Sanghavi DK, Franco PM, Gopal N, Lehman LM, Dong Y, Pickering BW, Herasevich V (2021). Are we ready for video recognition and computer vision in the intensive care Unit? A survey. Appl Clin Inform.

[29] Magi N, Prasad BG (2020). Activity monitoring for ICU patients using deep learning and image processing. SN COMPUT. SCI.

[30] Cahyono F, Wirawan W, Rachmadi RF (2020). Face recognition system using facenet algorithm for employee presence.

[31] Baltrusaitis T, Zadeh A, Lim YC, Morency LP (2018). Openface 2.0: Facial behavior analysis toolkit.

[32] Wood E, Baltruaitis T, Zhang X, Sugano Y, Robinson P, Bulling A (2015). Rendering of eyes for eye-shape registration and gaze estimatio.

[33] Cao Z, Hidalgo G, Simon T, Wei S, Sheikh Y (2021). OpenPose: realtime multi-person 2D pose estimation using part affinity fields. IEEE Trans Pattern Anal Mach Intell.

[34] Halladay CW, Sillner AY, Rudolph JL (2018). Performance of electronic prediction rules for prevalent delirium at hospital admission. JAMA Netw Open.

[35] Corradi JP, Thompson S, Mather JF, Waszynski CM, Dicks RS (2018). Prediction of incident delirium using a random forest classifier. J Med Syst.

[36] Coombes CE, Coombes KR, Fareed N (2021). A novel model to label delirium in an intensive care unit from clinician actions. BMC Med Inform Decis Mak.

[37] Herasevich V (2011). ICU data mart: a non-iT approach. A team of clinicians, researchers and informatics personnel at the Mayo Clinic have taken a homegrown approach to building an ICU data mart. Healthcare informatics: the business magazine for information and communication systems.

[38] Jones AE, Trzeciak S, Kline JA (2009). The sequential organ failure assessment score for predicting outcome in patients with severe sepsis and evidence of hypoperfusion at the time of emergency department presentation. Crit Care Med.

[39] Romero-Brufau S, Gaines K, Nicolas CT, Johnson MG, Hickman J, Huddleston JM (2019). The fifth vital sign? Nurse worry predicts inpatient deterioration within 24 hours. JAMIA Open.

[40] Ely EW, Margolin R, Francis J, May L, Truman B, Dittus R, Speroff T, Gautam S, Bernard GR, Inouye SK (2001). Evaluation of delirium in critically ill patients: validation of the confusion assessment method for the intensive care unit (CAM-ICU). Crit Care Med.

[41] Ely EW, Inouye SK, Bernard GR, Gordon S, Francis J, May L, Truman B, Speroff T, Gautam S, Margolin R, Hart RP, Dittus R (2001). Delirium in mechanically ventilated patients: validity and reliability of the confusion assessment method for the intensive care unit (CAM-ICU). JAMA.

[42] Krewulak KD, Rosgen BK, Ely EW, Stelfox HT, Fiest KM (2020). The CAM-ICU-7 and ICDSC as measures of delirium severity in critically ill adult patients. PLoS One.

[43] Ho M, Montgomery A, Traynor V, Chang C, Kuo KN, Chang H(, Chen K (2020). Diagnostic performance of delirium assessment tools in critically ill patients: A systematic review and meta-analysis. Worldviews Evid Based Nurs.

[44] Khan B, Perkins AJ, Gao S, Hui SL, Campbell NL, Farber MO, Chlan LL, Boustani MA (2017). The confusion assessment method for the ICU-7 delirium severity scale: A novel delirium severity instrument for use in the ICU. Crit Care Med.

[45] Jones RN, Cizginer S, Pavlech L, Albuquerque A, Daiello LA, Dharmarajan K, Gleason LJ, Helfand B, Massimo L, Oh E, Okereke OI, Tabloski P, Rabin LA, Yue J, Marcantonio ER, Fong TG, Hshieh TT, Metzger ED, Erickson K, Schmitt EM, Inouye SK, Better Assessment of Illness (BASIL) Study Group (2019). Assessment of instruments for measurement of delirium severity: A systematic review. JAMA Intern Med.

[46] Trzepacz P (2001). Validation of the delirium rating scale-revised-98: comparison with the delirium rating scale and the cognitive test for delirium. J Neuropsychiatry Clin Neurosci.

[47] Khan BA, Perkins AJ, Campbell NL, Gao S, Farber MO, Wang S, Khan SH, Zarzaur BL, Boustani MA (2019). Pharmacological management of delirium in the intensive care unit: A randomized pragmatic clinical trial. J Am Geriatr Soc.

[48] Vasunilashorn SM, Schulman-Green D, Tommet D, Fong T, Hshieh T, Marcantonio E, Metzger E, Schmitt E, Tabloski P, Travison T, Gou Y, Helfand B, Inouye S, Jones R, the BASIL Study Team (2020). New delirium severity indicators: generation and internal validation in the better assessment of illness (BASIL) study. Dement Geriatr Cogn Disord.

[49] Lewis JR (2018). The system usability scale: past, present, and future. International Journal of Human–Computer Interaction.

[50] Sidani S, Epstein DR, Bootzin RR, Moritz P, Miranda J (2009). Assessment of preferences for treatment: validation of a measure. Res Nurs Health.

[51] Pun BT (2019). Caring for critically ill patients with the ABCDEF bundle: results of the ICU liberation collaborative in over 15,000 adults. Critical care medicine.

[52] Stollings JL, Kotfis K, Chanques G, Pun BT, Pandharipande PP, Ely EW (2021). Delirium in critical illness: clinical manifestations, outcomes, and management. Intensive Care Med.

[53] Girard TD, Ely EW, MIND-USA Investigators (2019). Haloperidol and ziprasidone for treatment of delirium in critical illness. Reply. N Engl J Med.

[54] Shinde S, Kothari A, Gupta V (2018). YOLO based human action recognition and localization. Procedia Computer Science.

[55] Ruopp MD, Perkins NJ, Whitcomb BW, Schisterman EF (2008). Youden index and optimal cut-point estimated from observations affected by a lower limit of detection. Biom J.

[56] Miranda F, Gonzalez F, Plana MN, Zamora J, Quinn TJ, Seron P (2023). Confusion assessment method for the intensive care unit (CAM-ICU) for the diagnosis of delirium in adults in critical care settings. Cochrane Database Syst Rev.

[57] Fong TG, Inouye SK (2022). The inter-relationship between delirium and dementia: the importance of delirium prevention. Nat Rev Neurol.

[58] Fong TG, Davis D, Growdon ME, Albuquerque A, Inouye SK (2015). The interface between delirium and dementia in elderly adults. Lancet Neurol.

[59] Gross AL, Jones RN, Habtemariam DA, Fong TG, Tommet D, Quach L, Schmitt E, Yap L, Inouye SK (2012). Delirium and long-term cognitive trajectory among persons with dementia. Arch Intern Med.

[60] Fick DM, Steis MR, Waller JL, Inouye SK (2013). Delirium superimposed on dementia is associated with prolonged length of stay and poor outcomes in hospitalized older adults. J Hosp Med.

[61] Nitchingham A, Caplan GA (2021). Current challenges in the recognition and management of delirium superimposed on dementia. Neuropsychiatr Dis Treat.

[62] Yeung S, Downing NL, Fei-Fei L, Milstein A (2018). Bedside computer vision - moving artificial intelligence from driver assistance to patient safety. N Engl J Med.

[63] Saner H, Schütz N, Botros A, Urwyler P, Buluschek P, du Pasquier G, Nef T (2020). Potential of ambient sensor systems for early detection of health problems in older adults. Front Cardiovasc Med.

